# The Yeast *Saccharomyces cerevisiae* as a Model to Study the Anti-Aging Activity of Phycocyanin

**DOI:** 10.3390/ijms27020960

**Published:** 2026-01-18

**Authors:** Donata Cassamagnaghi, Stefania Citterio, Enzo Martegani, Sonia Colombo

**Affiliations:** Department of Biotechnology and Biosciences, University of Milano-Bicocca, Piazza Della Scienza 2, 20126 Milan, Italy; d.cassamagnaghi@gmail.com (D.C.); stefania.citterio@unimib.it (S.C.); enzo.martegani@unimib.it (E.M.)

**Keywords:** ROS, aging, chronological life span, yeast, *S. cerevisiae*, nutrient sensing, *A. platensis*, spirulina

## Abstract

We recently published that phycocyanin, a phycobiliprotein which accounts for up to 20% of *Arthrospira platensis* dry weight, has a powerful anti-aging effect, greatly extending the chronological life span (CLS) of yeast cells grown in synthetic-defined medium, both under caloric restriction (CR) conditions (0.2% glucose) or under non-CR conditions (2% glucose). In this study, to explore the molecular mechanisms underlying the effects of phycocyanin, we investigated its impact on key signaling pathways involved in aging. Specifically, we performed CLS experiments using *ras2Δ* and *snf1Δ* yeast mutants. The Snf1 pathway is known to promote longevity (anti-aging), whereas the Ras2/PKA pathway accelerates aging (pro-aging). We show that, while in the *snf1Δ* mutant the anti-aging effect of phycocyanin was still evident, in the *ras2Δ* mutant, phycocyanin did not appear to exert any anti-aging activity, leading us to hypothesize that the Ras2/PKA pathway may be essential to mediate the anti-aging effect of phycocyanin. To evaluate the activity of phycocyanin under different nutritional conditions, we performed the CLS experiment in a YPDA-rich medium. We show that in this medium, phycocyanin accelerated the chronological aging process of yeast cells, greatly decreasing the CLS, both when glucose was present at low (0.2%) or at high (2%) concentration. Our data suggest that *Saccharomyces cerevisiae* could serve as a model not only to investigate the anti-aging properties and targets of phycocyanin, but also its potential side effects, which are possibly present in higher eukaryotes under certain conditions.

## 1. Introduction

Aging is a complex and inevitable process marked by a progressive decline in cellular and tissue function. This deterioration increases the risk of developing chronic diseases, including cancer, cardiovascular conditions, and neurodegenerative disorders. [1,2]. In recent years, there has been growing attention to develop strategies aimed at counteracting the effects of aging, with particular interest in marine-derived compounds [3]. In this context, *Arthrospira platensis*, more commonly known as *Spirulina*, has attracted growing scientific and commercial interest. This cyanobacterium is an exceptionally rich source of bioactive compounds, many of which are already known for their potential health-promoting effects, while others have yet to be fully identified and characterized. Phycocyanin is a complex composed of proteins belonging to the phycobiliprotein family and water-soluble pigments of photosynthesis, the phycocyanobilins, and it is particularly abundant in the cyanobacterium *A. platensis*, constituting approximately 20% of its dry weight [4]. Phycocyanin is an oligomeric protein composed of α and β subunits with a molecular weight of 16.3 kD and 18.9 kD, respectively [4]. Several scientific studies suggest that *A. platensis* may have various applications in both medical and food industries [5], and phycocyanin is incorporated into diets in various forms such as powders, tablets, extracts, and supplements. In particular, phycocyanin is believed to play a key role in the anti-cancer, anti-inflammatory, and antioxidant effects of *A. platensis* [6,7,8]. It has also been demonstrated that phycocyanin reduces oxidative stress in both yeast and mammalian systems [9,10].

Recently we investigated the effect of phycocyanin on the survival of *Saccharomyces cerevisiae* cells growing in synthetic-defined medium and showed that phycocyanin has proven to possess anti-aging properties, greatly extending the chronological life span of yeast cells regardless of whether the glucose was present at a low (0.2%) or high (2%) concentration [11]. Aging, together with its wide range of age-associated conditions, is a complex, multifactorial process influenced by both genetic and environmental factors, involving many interconnected pathways and regulatory networks. In this regard, the yeast *Saccharomyces cerevisiae* serves as a valuable model organism for studying eukaryotic cells and offers important insights into the biology of human aging. This is because the key molecular pathways governing aging are evolutionarily conserved between yeast and higher eukaryotes [12,13,14,15,16,17]. In this study, we examined how phycocyanin influences key signaling pathways associated with aging in yeast to understand the molecular mechanisms behind its anti-aging properties. We show data leading us to hypothesize that the Ras2/PKA pathway may be essential to mediate the anti-aging effect of phycocyanin. The composition of the culture medium in which *Saccharomyces cerevisiae* is grown is a key factor influencing multiple cellular processes, including growth and proliferation, stress resistance, and chronological aging [18,19,20,21,22,23]. To explore how the medium composition influences the chronological aging process, we performed the chronological aging experiments in YPDA medium, which is particularly rich in nutrients (amino acids, peptides, nucleosides, etc.). We show that in rich medium, unlike what happens in synthetic-defined medium, phycocyanin accelerated the chronological aging process of yeast cells, leading to a marked reduction in their chronological life span, indicating that *S. cerevisiae* may be a valuable model not only for studying the anti-aging effects and molecular targets of phycocyanin, but also its potential side effects, possibly present in higher eukaryotic systems under certain conditions.

## 2. Results and Discussion

### 2.1. Effect of Phycocyanin on Saccharomyces cerevisiae snf1Δ and ras2Δ Mutants

**Figure 1:**
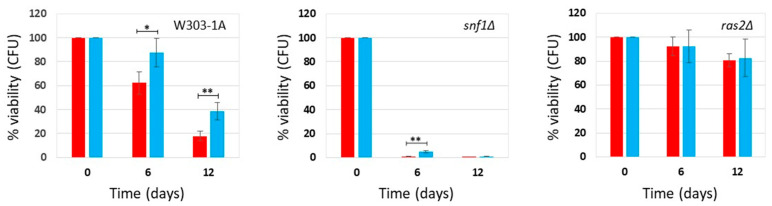
Cell survival in W303-1A, *snf1Δ*, and *ras2Δ* cells grown in SD medium containing 0.2% glucose in the presence and absence of phycocyanin (given at the moment of inoculation). Cell viability of W303-1A, *snf1Δ*, and *ras2Δ* cells, either untreated (red) or treated (blue) with 4.3 mg/mL (±0.1 mg/mL) phycocyanin, was analyzed by measuring colony-forming units (CFUs) after 3 days of growth at 30 °C. Cell survival is expressed as % to the CFUs at time zero. The means of 3 independent experiments with standard deviations are reported. Student’s *t*-test * *p* < 0.05 and ** *p* < 0.01.

**Figure 2:**
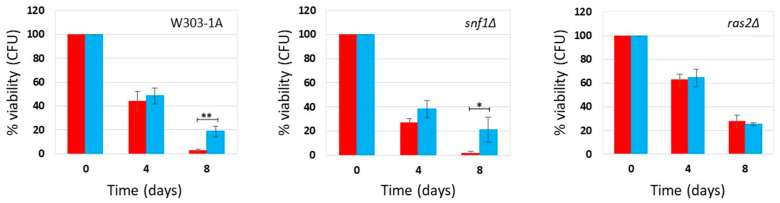
Cell survival in W303-1A, *snf1Δ*, and *ras2Δ* cells grown in SD medium containing 2% glucose in the presence and absence of phycocyanin (given at the moment of inoculation). Cell viability of W303-1A, *snf1Δ*, and *ras2Δ* cells, either untreated (red) or treated (blue) with 3.9 mg/mL (±0.4 mg/mL) phycocyanin, was analyzed by measuring colony-forming units (CFUs) after 3 days of growth at 30 °C. Cell survival is expressed as % to the CFUs at time zero. The means of 3 independent experiments with standard deviations are reported. Student’s *t*-test * *p* < 0.05 and ** *p* < 0.01.

*Saccharomyces cerevisiae*, commonly known as budding yeast, is a key model organism for studying eukaryotic cells and offers valuable insights into the biological mechanisms underlying human aging [13,14]. We recently demonstrated that phycocyanin exerts a potent anti-aging effect by significantly extending the chronological lifespan (CLS) of yeast cells cultured in synthetic-defined medium (SD medium), under both calorie restriction (CR) conditions (0.2% glucose) or non-CR conditions (2% glucose) [11]. In this study, we compared the survival of the wild-type strain and two mutant strains (*snf1Δ* and *ras2Δ* mutants) to explore the influence of phycocyanin on signal transduction pathways associated with longevity and to identify its physiologically relevant targets. The Snf1/AMPK pathway (sucrose-non-fermenting/AMP-activated protein kinase) is an anti-aging pathway [24], while the Ras2/PKA (Rat sarcoma/protein kinase A) pathway is a pro-aging one [25,26]. We inoculated wild-type cells and the *snf1Δ* and *ras2Δ* mutants in SD medium containing 0.2% glucose or 2% glucose, in the absence and presence of phycocyanin (for details about the phycocyanin used in this study, see the Materials and Methods section), respectively, 4.3 mg/mL (±0.1 mg/mL) and 3.9 mg/mL (±0.4 mg/mL) (day 3 of experiment). When the carbon source present in the culture medium is completely consumed, cells enter the stationary phase, do not carry out any cell division, and reach the maximum cell concentration (day 0 CLS). We monitored the progressive loss of viability of these stationary phase cells kept in their culture medium from day 0 until day 12 (SD medium containing 0.2% glucose) or day 8 (SD medium containing 2% glucose) and used them as an index of the chronological aging process [27] (Figure 1 and Figure 2). In the wild-type strain grown under conditions of caloric restriction (0.2% glucose), phycocyanin showed a potent anti-aging effect, confirming previously published data [11]. Cell survival increased significantly on day 6 of CLS, and on day 12, the viability of cells treated with phycocyanin was approximately double that of untreated cells (Figure 1). The anti-aging effect of phycocyanin was also clear in the *snf1Δ* strain (Figure 1). In particular, on day 6 of CLS, all cells in the untreated sample were non-viable, while the presence of phycocyanin allowed the survival of a small fraction of cells. Furthermore, as expected, the *snf1Δ* strain was less long-lived than the wild-type strain (Figure 1). The *ras2Δ* strain, known for being longer-lived compared to the wild-type strain [28], as also confirmed by our data (Figure 1), did not show significant differences between phycocyanin-treated and untreated samples (Figure 1). At both time points (days 6 and 12), the survival rate was indeed comparable. As previously published [11], phycocyanin also showed a potent anti-aging activity when the wild-type strain was grown in SD medium containing 2% glucose (non-caloric restriction conditions) (Figure 2). Moreover, as expected, in this strain grown in the absence of phycocyanin, cell survival was lower than in CR conditions (Figure 1 and Figure 2). Like in the wild-type strain, also in the *snf1Δ* strain, the presence of phycocyanin increased cell survival, demonstrating an anti-aging activity in this mutant (Figure 2). Furthermore, in the absence of phycocyanin, cell survival was higher than that observed under CR conditions (Figure 1 and Figure 2), consistent with the known role of the Snf1 protein being involved in metabolic pathways activated under conditions of low glucose availability [24]. Finally, in the *ras2Δ* strain, phycocyanin also did not appear to exert any anti-aging activity (Figure 2) in this nutritional condition. This data suggests that the Ras2/PKA pathway may be essential to mediate the anti-aging effect of phycocyanin, leading us to hypothesize a possible direct involvement of this signal transduction pathway in the molecular mechanism of action of this compound. Furthermore, as expected, the *ras2Δ* strain was longer-lived than the wild-type strain (Figure 2).

### 2.2. Effect of Phycocyanin on Reactive Oxygen Species Accumulation in snf1Δ and ras2Δ Mutants

**Figure 3:**
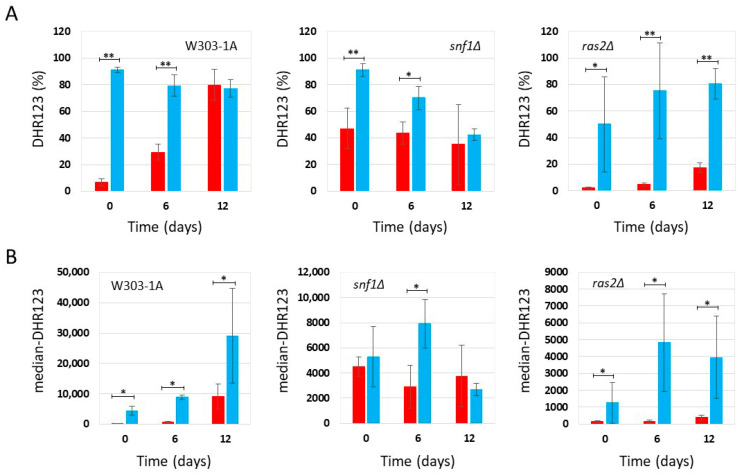
Flow cytometric analysis of W303-1A, *snf1Δ*, and *ras2Δ* cells grown in synthetic medium containing 0,2% glucose, either untreated (red bars) or treated (blue bars) with 4.3 mg/mL (±0.1 mg/mL) phycocyanin. Dihydrorhodamine 123 (DHR123) was used to assay ROS accumulation (for more details, see Supplementary Figures S1 and S2). (**A**) Percentage of cells positive for DHR123 staining. The means of 3 independent experiments with standard deviations are reported. Student’s *t*-test * *p* < 0.05 and ** *p* < 0.01. (**B**) Median fluorescence intensity (median-DHR123). The means of 3 independent experiments with standard deviations are reported. Mann–Whitney U test * *p* < 0.05.

Since oxidative stress and the accumulation of reactive oxygen species (ROS) play a key role in aging [29], we determined the accumulation of ROS in *snf1Δ* and *ras2Δ* mutants in the context of CLS experiments. Therefore, as described previously (see Figure 1 and Figure 2), the presence of phycocyanin in the medium significantly increased survival of wild-type and *snf1Δ* cells, while in the *ras2Δ* strain, phycocyanin did not appear to exert any anti-aging activity. In parallel, using a cytofluorimeter, we determined the accumulation of ROS by staining the chronologically aged cells with dihydrorhodamine123 (DHR123) (Supplementary Figure S1). Flow cytometry data were analyzed using CytExpert(version 2.6) and Floreada.io software. The results obtained from the cytofluorimetric analysis conducted on the wild-type strain grown under conditions of caloric restriction (0.2% glucose) confirmed the data recently published by Nova et al. [11]. In particular, we observed that wild-type cells treated with phycocyanin, although more viable than untreated controls (Figure 1), exhibited a higher percentage of DHR123-positive cells (Figure 3A) and an increased median fluorescence intensity (median-DHR123) within the population (Figure 3B). These findings are consistent with previously published data [11] and suggest that phycocyanin may exert a protective effect by promoting cell survival through elevated ROS levels. It can be hypothesized that this increase in ROS triggers an adaptive response to oxidative and other forms of stress, known as hormesis, which renders cells more resistant to subsequent, otherwise harmful, exposures to the same stressor [30,31]. Similarly to what was observed for wild-type cells, *snf1Δ* cells treated with phycocyanin —although longer-lived than the untreated ones (Figure 1)—also displayed a higher percentage of DHR123-positive cells (Figure 3A) and an increased median fluorescence intensity (median-DHR123) within the population (Figure 3B) compared to untreated cells. These findings suggest that, in this strain as well, phycocyanin may promote cell survival through hormesis-related mechanisms. Finally, in *ras2Δ* cells, although no differences in cell viability were observed between samples treated or untreated with phycocyanin (Figure 1), treatment with this compound also led to an increased percentage of DHR123-positive cells and a higher median fluorescence intensity (median-DHR123) within the population (Figure 3A,B).

**Figure 4:**
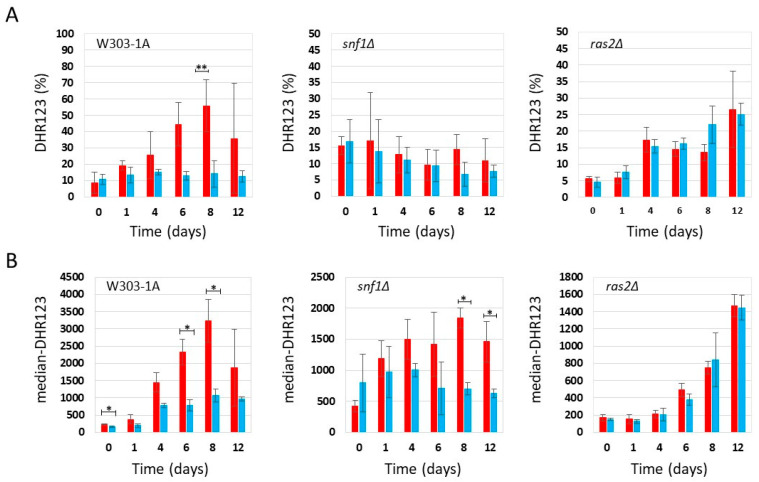
Flow cytometric analysis of W303-1A, *snf1Δ*, and *ras2Δ* cells grown in synthetic medium containing 2% glucose, either untreated (red bars) or treated (blue bars) with 3.9 mg/mL (±0.4 mg/mL) phycocyanin. Dihydrorhodamine 123 (DHR123) was used to assay ROS accumulation (for more details, see Supplementary Figures S1 and S2). (**A**) Percentage of cells positive for DHR123 staining. The means of 3 independent experiments with standard deviations are reported. Student’s *t*-test ** *p* < 0.01. (**B**) Median fluorescence intensity (median-DHR123). The means of 3 independent experiments with standard deviations are reported. Mann–Whitney U test * *p* < 0.05.

Under non-CR conditions (2% glucose), flow cytometry analysis showed that wild-type cells treated with phycocyanin, which were more viable than untreated cells (Figure 2), exhibited a lower percentage of DHR123-positive cells (Figure 4A) and a reduced median fluorescence intensity (median-DHR123) (Figure 4B) compared with untreated cells. These findings suggest that under these conditions, phycocyanin may exert a protective effect and promote cell survival through a mechanism independent of hormesis. As observed in wild-type cells, *snf1Δ* cells treated with phycocyanin also exhibited greater longevity compared with untreated cells (Figure 2). In these cells, the percentage of DHR123-positive cells was similar to that found in untreated controls (Figure 4A). However, the median fluorescence intensity (median DHR123) within the population was lower than that of untreated cells (Figure 4B), suggesting that, as in the previous strain, phycocyanin may confer a protective effect and promote cell survival through a mechanism distinct from hormesis. Finally, in *ras2Δ* cells, no differences in viability were observed between samples treated and untreated with phycocyanin (Figure 2) and both the percentage of DHR123-positive cells and the median fluorescence intensity (median-DHR123) in the population between phycocyanin-treated and untreated cells was comparable (Figure 4A,B). Overall, our findings suggest that, in both the wild-type and the *snf1Δ* mutant, phycocyanin may exert a protective effect and may promote cell survival through hormesis when the cells are grown in SD medium containing 0.2% glucose. Conversely, in SD medium containing 2% glucose, phycocyanin appears to promote cell survival through a mechanism other than hormesis.

### 2.3. Phycocyanin Accelerates the Chronological Aging Process of Yeast Cells Grown in Rich Medium

**Figure 5:**
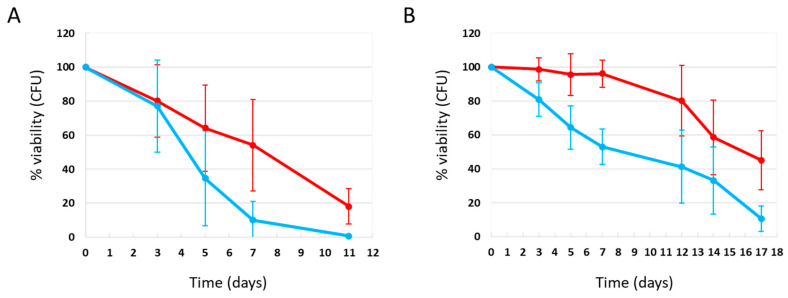
Cell survival in W303-1A cells grown in YPDA medium containing 0.2% glucose (**A**) and 2% glucose (**B**) in the presence and absence of phycocyanin (given at the moment of inoculation). Cell viability of W303-1A cells, either untreated (red) or treated (blue) with 4.6 mg/mL (±0.4 mg/mL) (**A**) and 4.6 mg/mL (±0.2 mg/mL) (**B**) phycocyanin, was analyzed by measuring colony-forming units (CFUs) after 3 days of growth at 30 °C. Cell survival is expressed as % to the CFUs at time zero. The means of 3 independent experiments with standard deviations are reported.

It is known that the composition of the culture medium in which *Saccharomyces cerevisiae* cells are grown plays a significant role in regulating various parameters, including growth and proliferation, stress tolerance, and chronological aging [18,19,20]. In particular, studies have shown that lifespan extension in *Saccharomyces cerevisiae* occurs not only when glucose levels in the growth medium are reduced, but also when the concentration of amino acids is lowered, suggesting that overall caloric reduction, rather than the limitation of a specific nutrient, is responsible for the increased longevity, in line with the well-documented effects of caloric restriction on aging in mammals [20]. In this context, Lee et al. [22] have shown that methionine restriction strongly extends lifespan across multiple experimental models, including yeast, Drosophila, rodents, and mammals. However, some amino acids, such as isoleucine, valine, and leucine, have been shown to extend the chronological lifespan of cells, even in the absence of autophagy, indicating that these amino acids function not only as nutrients but also as regulators of key metabolic and signaling pathways [18,23]. Finally, Wu et al. [21] have shown that caloric restriction confers an anti-aging effect only in the context of the SD medium. Based on these considerations, and to investigate the role of culture medium composition in regulating chronological aging in *Saccharomyces cerevisiae*, we performed the chronological aging experiments in YPDA medium, which is particularly rich in nutrients such as amino acids, peptides, nucleosides, etc. Yeast cells were grown in YPDA medium under CR (0.2% glucose) or non-CR (2% glucose) conditions, in the absence and presence of phycocyanin, respectively, 4.6 mg/mL (±0.4 mg/mL) and 4.6 mg/mL (±0.2 mg/mL) (day 3 of experiment), and their colony-forming unit (CFU) ability was measured over time. Our results showed that in rich medium, phycocyanin did not exhibit an anti-aging effect, instead significantly reducing their chronological lifespan at both low (0.2%) and high (2%) glucose concentrations (Figure 5).

As previously shown by Wu et al., cells grown in rich medium containing 2% glucose displayed increased longevity compared to those cultured in the same medium containing a lower percentage of glucose [21]. Here we show that this phenotype is independent of the presence or absence of phycocyanin, suggesting that caloric restriction confers an anti-aging effect only in the context of the SD medium. However, phycocyanin under these rich nutritional conditions behaves in an opposite way and reduces the chronological lifespan. Interestingly, hydroxycitric acid, a caloric restriction mimetic we previously studied [32], also accelerated aging in yeast cells grown in rich medium, having instead a protective effect on cells cultured in SD medium(unpublished data by Baroni et al. [33]). Taken together, our results comparing survival in SD and rich media support the notion that cellular longevity depends more on the proper balance of nutrients than on their absolute abundance. Imbalances in nutrients may induce metabolic stress, disrupt cellular homeostasis, and ultimately shorten CLS.

**Figure 6:**
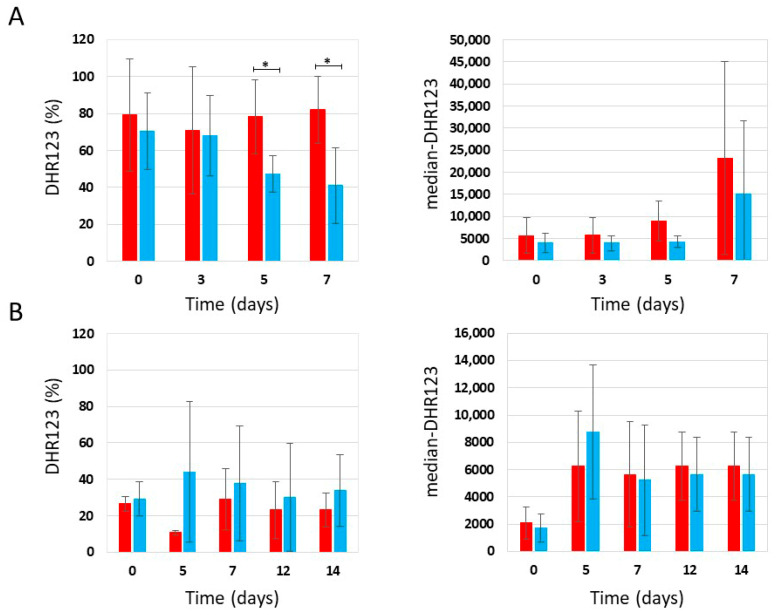
Flow cytometric analysis of W303-1A cells grown in YPDA medium containing 0,2% glucose (**A**) or 2% glucose (**B**), either untreated (red bars) or treated (blue bars) with 4.6 mg/mL (±0.4 mg/mL) (**A**) and 4.6 mg/mL (±0.2 mg/mL) (**B**) phycocyanin. Dihydrorhodamine 123 (DHR123) was used to assay ROS accumulation. Left panels: DHR123 (%); the means of 3 independent experiments with standard deviations are reported. Student’s *t*-test * *p* < 0.05. Right panels: Median fluorescence intensity (median-DHR123); the means of 3 independent experiments with standard deviations are reported. Mann–Whitney U test always gives *p* > 0.05.

In parallel with the CLS experiment, we determined the accumulation of reactive oxygen species by staining the chronologically aged cells with DHR123. Our results showed that in YPDA medium containing 0.2% glucose, phycocyanin-treated cells showed a lower percentage of DHR123-positive cells and a lower median fluorescence intensity (median-DHR123) in the population than untreated cells (Figure 6A), while in YPDA medium containing 2% glucose, no significant difference between treated and untreated cells was observed (Figure 6B).

## 3. Materials and Methods

### 3.1. Yeast Strains and Media

Strains used in this study: W303-1A (*MAT*a *ade*2-1 *can*1-100 *his*3-11,15 *leu*2-3112 *trp*1-1 *ura*3-1) [34]; W303-1A with *ras2::URA3* [32]; W303-1A with *snf1:HIS3* [35]. Synthetic-defined medium (SD) contained either 0.2% glucose or 2% glucose, 6.7 g/L YNB *w*/*o* amino acids (supplied by ForMedium™, Swaffham, UK) and the selective drop-out CSM (Complete Supplement Mixture, supplied By ForMedium™, Swaffham, UK)-HIS-LEU-TRP-URA; in addition, 100 mg/L adenine and 50 mg/L histidine, leucine, tryptophan, and uracil were added. The rich medium (YPDA) consisted of 1% (*w*/*v*) yeast extract, 2% (*w*/*v*) peptone, 100 mg/L adenine, and either 0.2% or 2% (*w*/*v*) glucose. YPDA plates contained 2% (*w*/*v*)1 glucose, 2% (*w*/*v*) peptone, 1% (*w*/*v*) yeast extract, 100 mg/L adenine, and 2% (*w*/*v*) agar. Culture density was measured with a Coulter Counter (Coulter mod. Z2) on mildly sonicated, diluted samples.

Organic Spirulina extract (phycocyanin powder) used in this study was generously supplied by Algavista (Chennai, India) and is the same source material utilized in our previous publication [11]. As reported in the certificate of analysis, the initial purity was higher than 40%. However, the filtration step described below enabled the recovery of phycocyanin with a high level of purity, as confirmed by the absorbance ratio A620/A280. The measured ratio of 3.99 falls within the range of 0.7 ≤ A620/A280 ≤ 3.9, which is characteristic of reagent-grade phycocyanin [36] (Supplementary Figure S3). Phycocyanin powder was dissolved in the proper medium (the concentration slightly varies among experiments) and sterilized by filtration through 0.22 μm PES filters (Biosigma, Venice, Italy). Given the difficulty in filtering the culture medium containing phycocyanin and since a deposit of material on the filter was observed during the filtration step, as previously described, the absorbance at 620 nm and a calibration curve were used to determine whether phycocyanin loss occurred during this step and to determine the quantity of phycocyanin actually present in the medium [11]. On average, the concentration of phycocyanin after filtration was approximately 80% of the phycocyanin dissolved in the fresh medium. The pH of the fresh medium was 5.7 regardless of the presence of phycocyanin.

### 3.2. Aging Experiments and Cell Viability

We used one of the established procedures to measure chronological life span (CLS), as described by Fabrizio and Longo [27]. Assessment of clonogenicity using the CLS assay is the most commonly used method to quantify cell viability, which reflects a cell’s ability to divide. It should be emphasized that compromised proliferation does not necessarily lead to cell death; however, the literature suggests that clonogenic capacity represents an excellent indicator of cell death in a wide range of contexts, i.e., it represents a valid approximation to quantify survival in yeast populations [37,38,39,40]. The yeast cells were grown in either SD medium or YPDA medium (containing either 0.2% glucose or 2% glucose) at 30 °C on a shaker at 160 rpm and prolonged incubation in their original exhaust medium. The chronological aging process was monitored after the start of the stationary phase (day 0 of CLS) as progressive loss of cell viability. In particular, cells were plated on YPDA agar plates in triplicate, and colony-forming units (CFUs) were used to monitor viable cells after 3 days of growth at 30° C.

### 3.3. Staining with Dihydrorhodamine 123 (DHR123)

ROS were detected with DHR123 (Sigma Aldrich, Milan Italy) essentially as described by Madeo et al. [41]. DHR123 is a non-fluorescent probe which easily enters live cells due to its neutral, reduced structure. When ROS oxidizes it, it becomes rhodamine 123, which is highly fluorescent. DHR123 was added directly to the culture medium at the final concentration of 5 µg/mL (from a 2.5 mg/mL stock solution in ethanol), and cells were incubated for 2 h at 30 ° C with shaking in the dark. At the end of this period, cells were diluted in 50 mM TrisHCl pH 7.5 to 10^7^ cell/mL and analyzed using a cytofluorimeter (CytoFLEX©, Beckman Coulter, Inc., Brea, CA, USA), with excitation and emission settings of 488 and 525–550 nm (filter FL 1-H). The detector gains and threshold settings were the same for all the experiments and are the following: SSC, 122; FSC, 149; filter FL 1-H, 50. Threshold: Automatic on FSC. A total of 20.000 events were acquired for each sample, and data were processed using CytExpert software (Version 2.6) (Beckman, Milan Italy). In all the experiments, we used unstained cells as a control. We used a single stain (DHR123), so no compensation settings were applied for the cytofluorimetric analysis. The gating was performed using the same unstained cells data for setting ROS-negative cells (Supplementary Figures S1 and S2). Floreada.io software (Version 3.1) (a free web-based tool for flow cytometry analysis; https://floreada.io/, accessed on 13 December 2025) was used to calculate the median fluorescence values.

### 3.4. Statistical Analysis

All the experiments were conducted at least in triplicate, and the mean and standard deviation were shown. Student’s *t*-test and the Mann–Whitney U test (a free web-based tool was used: https://www.statskingdom.com/170median_mann_whitney.html, accessed on 13 December 2025) were used for assessing the significance of the experimental data. The experimental data were analyzed with Excel TM.

## 4. Conclusions

Phycocyanin, a complex consisting of proteins belonging to the phycobiliprotein family and of a pigment, phycocyanobilin, that can account for up to 20% of *Spirulina’s* dry weight, is considered one of the main contributors to the functional properties attributed to this cyanobacterium: numerous studies report antioxidant, anti-inflammatory, and even anticancer activities, making it a highly promising candidate for nutraceutical, pharmacological, and biotechnological applications [6,7]. In this paper we used *Saccharomyces cerevisiae* as a model organism to study the anti-aging activity of phycocyanin purified from *A. platensis* (formerly *Spirulina platensis*).

Understanding the mechanism of action of molecules is crucial for several reasons. Identifying the specific biological targets they interact with helps explain the effects they produce. Furthermore, knowledge of the mechanism allows for better prediction of potential side effects and toxicity. In order to find physiologically relevant phycocyanin targets, we studied the effect of this compound in mutants affecting longevity, in particular, the *snf1∆* and the *ras2∆* mutants, and found that the Ras2/PKA pathway may be essential to mediate the anti-aging effect of phycocyanin, leading us to hypothesize a possible direct involvement of this signal transduction pathway in the molecular mechanism of action of phycocyanin. Since oxidative stress and the accumulation of reactive oxygen species are considered important factors causing aging, and since data in the literature attribute an antioxidant role to phycocyanin [6,7,29], in parallel with the chronological life span experiments, we determined the accumulation of reactive oxygen species by staining the chronologically aged cells with DHR123. Although further experiments will be needed to draw definitive conclusions, our findings suggest that, in both the wild-type and the *snf1∆* mutant, phycocyanin may exert a protective effect and may promote cell survival through hormesis [30,31], an adaptive response to a variety of stresses according to which low doses of ROS act as essential signaling molecules to promote metabolic health and longevity, when the cells are grown in SD medium containing 0.2% glucose. Conversely, in SD medium containing 2% glucose, phycocyanin appears to promote cell survival through a mechanism other than hormesis.

Moreover, we showed that growth conditions and nutrient sensing modulate the anti-aging activity of phycocyanin in budding yeast. In particular, we showed that phycocyanin has a powerful anti-aging effect, greatly extending the chronological life span of yeast cells grown in SD medium both under calorie restriction (CR) conditions (0.2% glucose) or non-CR conditions (2% glucose), while in rich medium (YPDA), phycocyanin does not exhibit anti-aging activity, instead significantly reducing yeast chronological lifespan at both low (0.2%) and high (2%) glucose concentrations. Additionally, our data support prior studies [21] demonstrating that cells grown in rich medium containing 2% glucose exhibit greater longevity than those cultured in the same medium containing a lower percentage of glucose. We further showed that this effect occurs regardless of the presence or absence of phycocyanin, indicating that caloric restriction promotes anti-aging benefits only within the context of the synthetic medium. Taken together, our results support the idea that nutrient balance, in addition to caloric restriction, is a key factor for longevity of yeast.

In summary, our findings suggest that the anti-aging action of phycocyanin is strongly dependent on the metabolism of *S. cerevisiae* and that this microorganism may represent a useful model organism to investigate the anti-aging properties and targets of substances, but also their potential side effects, which are possibly present in higher eukaryotes under certain conditions.

## Data Availability

The data that support the findings of this study are available in the Supplementary Figures of this article. The original unprocessed data (flow cytometry list mode files and Excel with the mean and median values) can be obtained by request to the corresponding author (sonia.colombo@unimib.it).

## Supplementary Materials

The following supporting information can be downloaded at https://www.mdpi.com/article/10.3390/ijms27020960/s1.
