# Supplementary material for: The Yeast Saccharomyces cerevisiae as a Model to Study the Anti-Aging Activity of Phycocyanin
Source: Int J Mol Sci. 2026 Jan 18;27(2):960. doi: 10.3390/ijms27020960 (PMC12842097; doi:10.3390/ijms27020960)
Supplement: Supplementary file 1 [file ijms-27-00960-s001.zip › ijms-4030216-supplementary.pdf]

Supplementary Figure S1

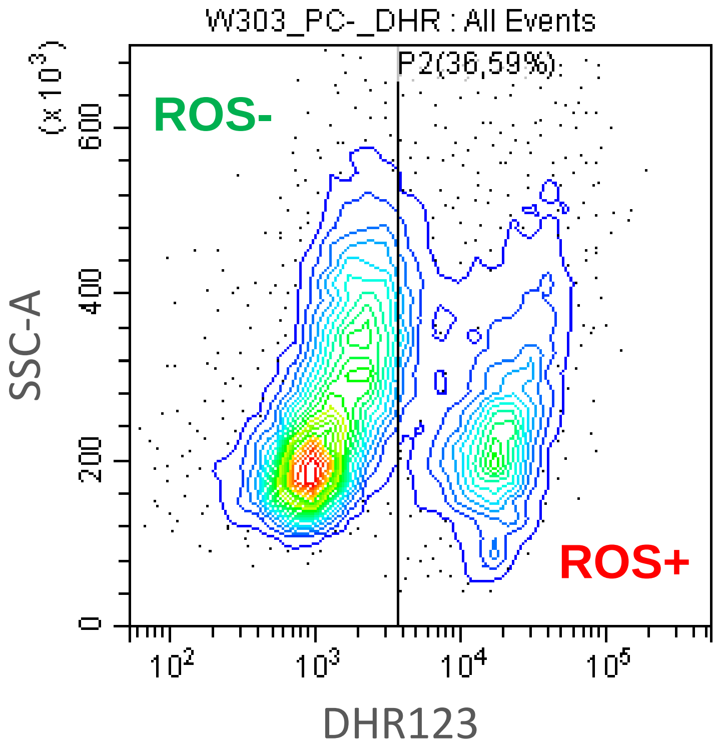

Supplementary Figure S1

Flow cytometry analysis of DHR123 stained yeast cells. DHR123 fluorescence is shown on the X-axis, while SSC-A (side scatter) is shown on the Y-axis. Cells in the left quadrant represent cells negative for DHR123 stain, while cells in the right quadrant represent cells positive for DHR123 stain.

Supplementary Figure S2

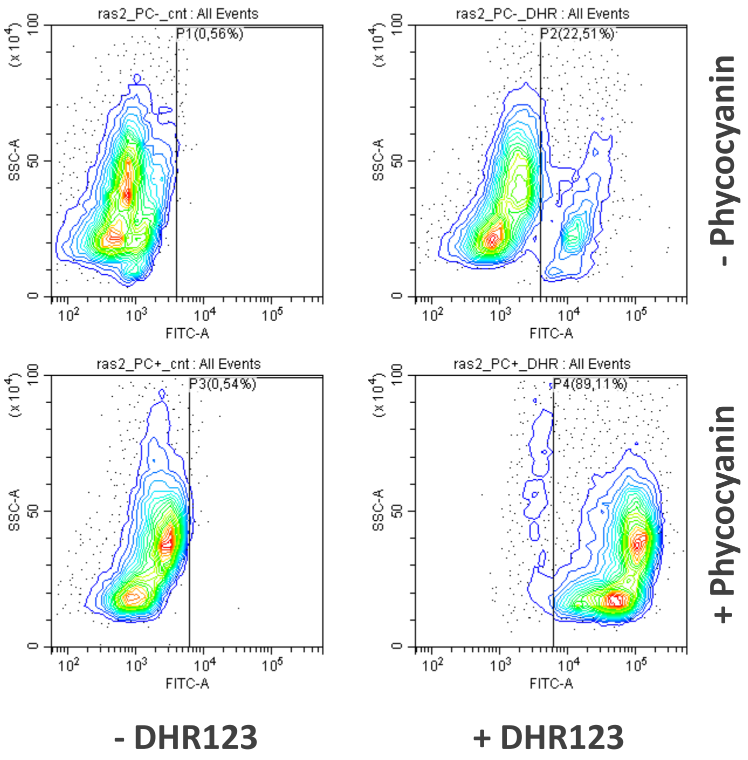

Supplementary Figure S2

Flow cytometry analysis of *ras2Δ* cells cultured in synthetic medium containing 0.2% glucose in the absence and presence of 4.3 mg/mL (+/-0.1 mg/ml) phycocycanin. Cells were stained with DHR123 as described in Material and Methods. A representative experiment is shown.

Supplementary Figure S3

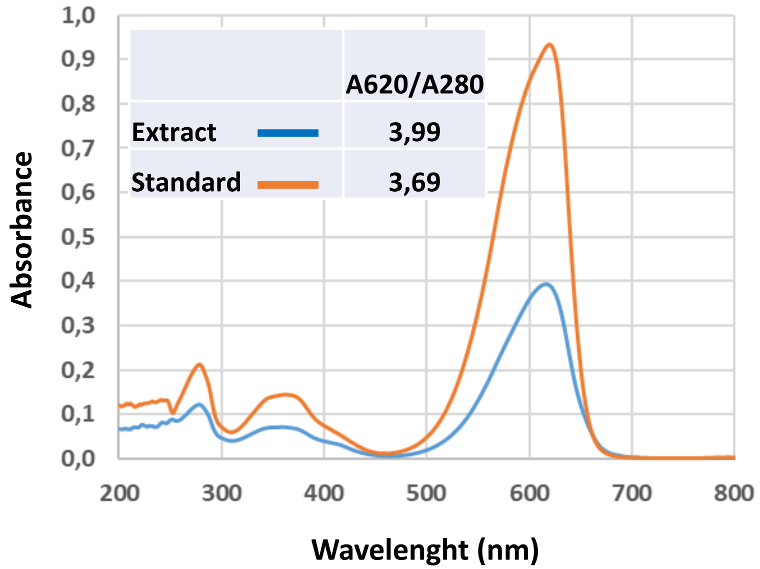

Supplementary Figure S3

Determination of the purity of phycocyanin used in this study. The degree of purity is determined by the A620/A280 ratio of samples prepared at a concentration of 100µg/mL in water. The absorption spectrum of the phycocyanin used in the study (Algavista, in blue) and of the Sigma commercial standard (in orange) is shown.
